# Poly-Lactide/Exfoliated C30B Interactions and Influence on Thermo-Mechanical Properties Due to Artificial Weathering

**DOI:** 10.3390/polym8040154

**Published:** 2016-04-20

**Authors:** Wendy Margarita Chávez-Montes, Guillermo González-Sánchez, Sergio Gabriel Flores-Gallardo

**Affiliations:** Centro de Investigación en Materiales Avanzados, S.C., Miguel de Cervantes No. 120, Complejo Industrial Chihuahua, Chihuahua 31000, Mexico; wendy.chavez@cimav.edu.mx (W.M.C.-M.); sergio.flores@cimav.edu.mx (S.G.F.-G.)

**Keywords:** PLA, nanocomposite, thermo-mechanical properties, biopolymer, artificial weathering

## Abstract

Thermal stability as well as enhanced mechanical properties of poly-lactide (PLA) can increase PLA applications for short-use products. The conjunction of adequate molecular weight (*M*_W_) as well as satisfactory thermo-mechanical properties, together, can lead to the achievement of suitable properties. However, PLA is susceptible to thermal degradation and thus an undesired decay of *M*_W_ and a decrease of its mechanical properties during processing. To avoid this PLA degradation, nanofiller is incorporated as reinforcement to increase its thermo-mechanical properties. There are many papers focusing on filler effects on the thermal stability and mechanical properties of PLA/nanocomposites; however, these investigations lack an explanation of polymer/filler interactions. We propose interactions between PLA and Cloisite30B (C30B) as nanofiller. We also study the effects on the thermal and mechanical properties due to molecular weight decay after exposure to artificial weathering. PLA blank and nanocomposites were subjected to three time treatments (0, 176, and 360 h) of exposure to artificial weathering in order to achieve comparable materials with different *M*_W_. *M*_W_ was acquired by means of Gel Permeation Chromatography (GPC). Thermo-mechanical properties were investigated through Thermogravimetric Analysis (TGA), Differential Scanning Calorimetry (DSC), X-ray Diffraction (XRD), Dynamic Mechanical Thermal Analysis (DMTA) and Fourier Transform Infrared Spectroscopy (FTIR).

## 1. Introduction

Biodegradable polymers are currently materials of considerable interest due to their advantages, such as being environmental benign and having an origin of renewable resources. Among the bio-based and biodegradable polymers, poly-lactide (PLA) is one of the popular choices as it is now available largely at commercial scale [1]. PLA is a biodegradable thermoplastic that has restricted applications due to its brittleness and poor crystallization behavior. Blending PLA with clay at the nanorange can develop an increase in toughness and thermal properties [2,3,4]. A solution largely developed over the past years has consisted of the incorporation of nanosized reinforcements within the polymer matrix, yielding so-called nanocomposite materials [5,6,7,8]. Various types of nanofillers have been considered as reinforcing agents throughout PLA matrix in order to enhance its thermo-mechanical properties as well as to provide additional functionalities like barrier properties [7,8,9], improved biodegradation rate [10], biomedical applications [11] and fire-resistance attributes [6]. Clays used in nanocomposites are those denominated as “organoclays”; cationic complexes in which the surface metal cations of natural clays have been exchanged with an organic cation surfactant. The surfactant layer is organophilic, allowing the inorganic clays to be dispersed in organic polymers [12]. One of the clays that have been used in many studies for the preparation of polymer/clay nanocomposites is Cloisite30B (C30B), which is the result of the organic modification of a sodium montmorillonite. C30B consists of several hundred individual plate-like structures with dimensions of 1 μm × 1 μm × 1 nm. These are held together by electrostatic forces. The uniform dispersion of C30B in PLA matrix is a general requirement for achieving improved mechanical and physical characteristics at the height of conventional plastics. Therefore, PLA is most likely to replace commodity plastics such as polyethylene (PE), polypropylene (PP), polystyrene (PS), and poly(ethylene terephthalate) (PET) [13,14,15]. However, to incorporate the C30B into a PLA matrix, it is often necessary to perform mixtures in molten state, which leads to a degradation of PLA and therefore molecular weight reduction. It has been observed that PLA is vulnerable to severe thermal degradation when it is processed above its melting temperature (especially above 180 °C). Thus, PLA processing undergoes heat transference and shear forces that results in a decrease of the molecular weight (*M*_W_) of PLA caused by depolymerization reactions. In addition to the latter, natural weathering (a condition to which polymers are exposed along their useful life) can also result in a *M*_W_ decay due to humidity and photo-oxidation depolymerization reactions [15]; however, these conditions can be simulated in the laboratory. An undesired *M*_W_ lowering of the final products results in poor mechanical performance and a reduction of thermal stability [13]. This unsatisfactory lack of heat stability and poor mechanical properties of PLA limits its applications [10], even when it was compounded to enhance them. According to Nieddu *et al.* [16], the combination of polymer and clays at the nanoscale, often results in remarkably improved mechanical and functional properties with respect to neat polymers or conventional composites, which leads to a higher Storage Modulus (*E*′). As the Modulus increases, the thermo-mechanical properties such as strength and heat resistance increase [13,17]. Although there have been several works studying the thermal and mechanical properties of PLA nanocompounded with clays, particularly Cloisite30B, the effect of molecular weight changes after artificial weathering exposure on the thermo-mechanical properties of nanocompounded PLA has not been sufficiently investigated. Since the molecular weight of polymers is one of the main factors affecting their thermal and mechanical properties, it is important to study the effect of molecular weight decay on the enhanced properties of nanocomposites, such as thermal resistance and elastic modulus. Additionally, interactions between PLA and nanoclay have not been explained. In this study, an organically modified layered montmorillonite-type C30B dispersed at nanorange throughout PLA matrix with different *M*_W_ due to artificial weathering was used in order to describe interactions between PLA and C30B, as well as the thermo-mechanical properties of PLA nanocomposites. In a previous work [18], we described the incorporation of C30B at the nanorange throughout PLA matrix, and so only the thermal and mechanical properties of these nanocomposites are presented in this paper.

## 2. Materials and Methods

PLA 2002D (semi-crystalline grade) was supplied by NatureWorks, Blair, NE, USA. PLA was stored in a dry, cool, and dark place before processing. Molecular weight distribution was determined by means of Gel Permeation Chromatography (GPC). An organomodified montmorillonite (MMT)-type Cloisite30B (C30B) was supplied by Southern Clay Products Inc. located in Gonzales, TX, USA and was used as filler. According to the manufacturer, the C30B was obtained by a modification of the natural MMT with a quaternary ammonium salt.

### 2.1. Preparation of PLA Nanocomposite and Blank

In a previous work [18], we studied the effect of artificial weathering on PLA filled with a nano-sized organomodified montmorillonite cloisite type C30B. After composite processing we found by means of X-ray diffraction (XRD) that the interplanar space on d_001_ increased due to the detachment of clay galleries. In addition, we studied the morphology of these composites by means of High Resolution-Transmission Electronic Microscopy (HR-TEM), and we could observe clay particles from single C30B platelet to eight stack platelets, both at nanorange. These results allowed us to conclude that clay particles’ size diminished until it reaches nanoscale. These nanocomposites were the subject of this study as a complementary investigation dealing with the explanation of PLA/exfoliated C30B interactions and their influence on thermo-mechanical properties due to artificial weathering. However, the preparation of PLA blank and nanocomposites are again described in this paper as follows. Blends of semi-crystalline PLA were carried out with a 5% load of C30B in order to obtain nanocomposites (named PLA/C30B) by a single-screw extruder Brabender Plasticorder DDRV752 (Brabender® GmbH & Co. KG, Kulturstr, Duisburg, Germany) with L/D ratio of 25:1, and a barrel of 19 mm in diameter. Mixtures were conducted with a temperature program of 150 °C in the feeding zone, and rose up to 180 °C in the die. The screw speed was set at 20 rpm. The mixtures were ground in a blender and introduced to a second extrusion stage under the same extruder conditions as previous in order to ensure a homogeneous material. Additionally, composites with dispersed filler at the nanoscale were obtained. The extrudate from the second stage was again ground. For the elaboration of flexion test probes, the ground extrudate from the second stage was placed into a Negri Bossi injection molding machine model V55-200 (New Castle, DE, USA) with mold temperature of 60 °C, and die temperature of 220 °C. PLA blank (PLA/B) was prepared with the same process as the nanocomposites but without the addition of C30B.

### 2.2. Obtaining Materials with Different Molecular Weights

In order to obtain materials with different molecular weights, the samples were degraded in an UV Artificial Weathering Tester (Q-Panel LabProducts Westlake, OH, USA) using a Fluorescent UVA-340 lamp with typical radiation of 0.77 W·m^−2^·nm^−1^, and 260–400 nm wavelength with peak intensity in 340 nm at 0, 176, and 360 h of exposure according to cycle 1 of the ASTM D5272 test method as follows: firstly, 8 h of UV exposure period at the black panel temperature of 60 ± 3 °C, followed by a condensation period of 4 h at the black panel temperature of 50 ± 3 °C. This cycle was repeated as many times as needed until completion of exposure time.

### 2.3. Characterization

*M*_W_ and polydispersity index (*M*_n_/*M*_W_) of the original PLA (PLA neat), the blank (PLA/B), and the nanocomposite (PLA/C30B), was determined using an Agilent 1200 Infinity Series Gel Permeation Chromatograph (GPC) (Santa Clara, CA, USA). High Performance Liquid Chromatography (HPLC)-grade Tetrahydrofuran (THF) was used as eluent with an average flow rate of 1 mL·min^−1^ at 40 °C. Polystyrene standards were used to calibrate the GPC. The samples were prepared at a concentration of 1 mg·mL^−1^ in THF. Thermogravimetric Analysis (TGA) was performed on a TA Instruments calorimeter SDTQ600 (New Castle, DE, USA) using an airflow rate equal to 50 mL·min^−1^. Samples (*ca.* 15 mg) were placed in an alumina can. PLA/B and PLA/C30B were heated from ambient temperature to 550 °C at a heating rate of 10 °C·min^−1^. Differential Scanning Calorimetry (DSC) tests were carried out using a TA Instruments Calorimeter DSC-Q 1000 (New Castle, DE, USA) under air atmosphere. Samples were heated at a scanning rate of 10 °C·min^−1^ from 30 to 200 °C. To investigate the thermal behavior of materials, Glass Transition Temperature (*T*_g_), Melting Temperature (*T*_m_), Melting Enthalpy (Δ*H*_m_), and Degree of Crystallinity (*X*_c_), were determined from a second heating cycle. XRD measurements were performed on an X’Pert Pro of PANalytical X-ray diffractometer (Westborough, MA, USA) using Cu Kα. The data was collected over a range of scattering angles (2 theta) of 4°–40° and a scan speed of 0.4 °·s^−1^. Storage Modulus (*E*′) and Loss Tangent (tan δ) were used as an indicator of the elastic modulus of the samples, and structural transformation was determined using a Dynamic Thermo-Mechanical Analyzer (DTMA) model RSAIII from TA Instruments (New Castle, DE, USA). For determination of the linear viscoelastic region (LVR), a strain sweep was carried out on each sample at 30 °C at a frequency of 1 Hz. Subsequently, a temperature sweep was performed from 30 to 90 °C at a scanning rate of 3 °C·min^−1^ under air atmosphere. The Attenuated Total Reflectance (ATR) by Fourier Transform Infrared (FTIR) spectra was recorded with a 4 cm^−1^ spectral resolution on a Perkin Elmer SpectrumGX spectrometer (Billerica, MA, USA) by signal-averaging 40 scans.

## 3. Results

### 3.1. Molecular Weight

Table 1 reports the *M*_W_ of PLA/B and PLA/C30B as a result of the exposure to artificial weathering; also, the *M*_W_ of neat PLA directly from the commercial pellets is reported.

Number Average Molecular Weight (*M*_n_) decay of PLA neat with respect to PLA/B and PLA/C30B at 0 h of exposure to artificial weathering was attributed to processing (extrusion and injection molding) of materials. Extrusion and injection molding of thermoplastics is a high-shear process that is performed in molten state. The effect of high shear rates and temperatures resulted in *M*_W_ decay in both PLA blank and nanocomposite. It could be observed that Weight Average Molecular Weight (*M*_W_) and *M*_n_ in PLA/B and PLA/C30B showed a significant reduction as the exposure to artificial weathering was increased. This suggests a depolymerization of PLA chains, which occurs as a result of thermal scission, humidity, and photo-oxidation to which the samples were subjected. Original PLA had a *M*_n_ of 135.9 kg·mol^−1^. After processing, PLA/B and PLA/C30B *M*_n_ values were 49.0 and 43.5 kg·mol^−1^, respectively. When samples were exposed to artificial weathering, *M*_n_ decreased to values of 16.4 kg·mol^−1^ for PLA/B and 11.3 kg·mol^−1^ for PLA/C30B, both at maximum exposure time (360 h) to artificial weathering. These results clearly showed first the degradation during processing and second the photodegradation and hydrolysis of PLA during artificial weathering. It has been reported that artificial weathering occurs in the manner of chain scission [17,19]. Solarski *et al.* [20] examined the effects of ageing of neat PLA and PLA nanocomposite. They concluded that mechanical and thermal properties of PLA underwent strong modifications due artificial weathering. They also observed faster degradation in PLA nanocomposites.

### 3.2. Thermo-Mechanical Properties

Filler/polymer interactions were described by a change in the thermal and mechanical properties of PLA/C30B compared with PLA/B. This change of thermo-mechanical properties was related to a reduction of the *M*_W_ of the studied materials. The effect of *M*_W_ on the thermo-mechanical properties of PLA blank and nanocomposite was studied by means of TGA, DSC, and DTMA.

#### 3.2.1. Thermogravimetric Analysis (TGA)

**Figure 1:**
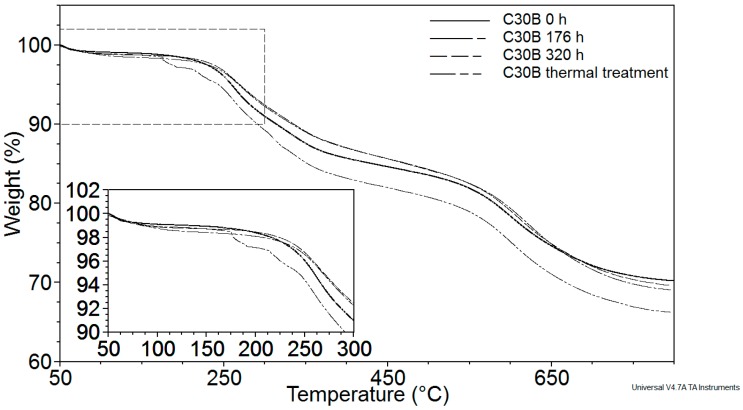
Thermogravimetric analysis of C30B exposed to both thermal treatment and artificial weathering.

Thermo-gravimetric analysis in the literature [12] has shown that the organic component of organoclays begins to break down at temperatures *ca.* 180 °C under non-oxidative environments, and significant degradation occurs just above this temperature. These degradation temperatures may be exceeded during melt processing of PLA. Therefore, it is of prime importance to study the thermal stability of the organic modifier and its implications for nanocomposite processing and properties. Figure 1 shows the thermogravimetric analysis of C30B. In order to observe the weight loss of the organomodifier during nanocomposite processing, a thermo-gravimetric (TG) curve from 30 to 800 °C at a heating rate of 10 °C·min^−1^ was performed, with two thermal treatments of C30B carried out at 180 °C for 10 min followed by another isotherm at 220 °C for 2 min, which correspond to the extrusion process followed by injection molding. The results showed that the weight loss of organomodifier was negligible (2.52%). We concluded that the content of organomodifier of C30B (amounting to about one third of the total mass) remains available to allow interactions with PLA. In addition, C30B alone was subjected to the same three exposure times of artificial weathering (0, 176, and 360 h) of blank and nanocomposites to observe a possible degradation of organomodifier due artificial weathering. However, from TGA measurements, the remaining organomodifier content in all the samples at the three exposure times to artificial weathering was *ca.* 30%. In addition, the onset of degradation of C30B was shown to occur at *ca.* 180 °C and the loss of decomposition products took place in two steps at 180 °C and *ca.* 400 °C. According to Filippi S. *et al.* [21], the first event of weight loss is due to degradation of the unconfined organomodifier that did not take part in the cation exchange reaction during synthesis. The presence of unreacted organomodifier on the surface of the organoclay particles was ascribed to improper washing during manufacture.

**Figure 2:**
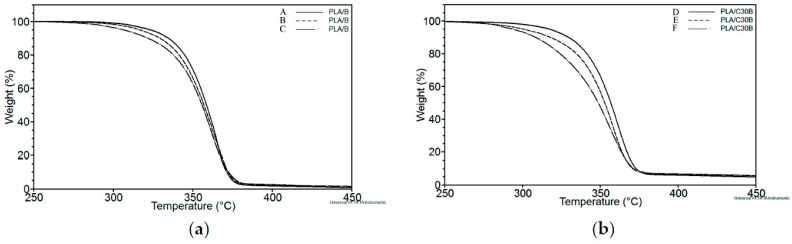
Thermogravimetric analysis (TGA) of (**a**) PLA/B (*M*_n_ in kg·mol^−1^ of A = 49.0, B = 33.1, and C = 16.4) and; (**b**) PLA/C30B (*M*_n_ in kg·mol^−1^ of D = 43.5, E = 31.9 and F = 11.3).

The weight loss curve shows the loss of mass at a constant heating rate of PLA/B (*M*_n_ in kg·mol^−1^ of A = 49.04, B = 33.06, C = 16.40) in Figure 2a and PLA/C30B (*M*_n_ in kg·mol^−1^ of D = 43.49, E = 31.90, F = 11.32) in Figure 2b. As can be seen from the plot, the decomposition process of PLA begins at about 300 °C and proceeds rapidly as the temperature increases up to 400 °C for all samples. It was also observed that the decomposition of PLA underwent a shift of sigmoidal curve shape to lower temperatures when *M*_n_ decreased, in both PLA/B and PLA/C30B. This can be attributed to the size of chains. Smaller PLA chains have fewer bonds to break down than larger PLA chains; therefore, it requires less energy to decompose PLA into CO_2_ and water [13]. PLA with the lowest *M*_n_ (Figure 2aC,bF) showed more pronounced weight loss decay on temperatures between *ca.* 280 to 350 °C. Degradation of PLA chains starts when the covalent bonds between atoms begin to split apart. Therefore, *M*_n_ decreased due to breakage of PLA chains until it transformed to CO_2_ and water. From Figure 2b, a residue at *ca.* 95% of weight loss can be seen, which corresponds to the quantity (5% weight residue) of C30B used as filler to obtain nanocomposites. Furthermore, even though a sigmoidal TG curve shape shift was observed, the temperature at which all samples were completely degraded was the same in both PLA/B and PLA/C30B, even when they had different *M*_n_.

Data of the decomposition temperatures from *T*_10%_ to *T*_90%_ of weight loss are shown in Table 2. *T*_10%_ of PLA/C30B shifted down *ca.* 6 °C in comparison to PLA/B, both with the highest *M*_n_ (without exposure to artificial weathering). This means that thermal degradation of PLA nanocomposites starts earlier than those obtained with the same processing but without the addition of C30B. Similarily, PLA/C30B with the lowest *M*_n_ showed a decrease of *T*_10%_ from about 21 °C in comparison to PLA/C30B with the highest *M*_n_. This indicates that chain breakage in PLA nanocomposites with shorter chains starts earlier than nanocomposites with bigger chains. Additionally, it was observed that *T*_90%_ of PLA/B showed a small difference of *ca.* 1 °C in samples with the highest, medium, and lowest *M*_n_. This behavior resembles *T*_90%_ of PLA/C30B with a variance of *ca.* 3 °C in different *M*_n_. Thermal degradation resistance of PLA matrix was not improved by the presence of C30B in comparison with PLA/B. This effect was also reported by Pandey *et al.* [22] in their research, where they showed that PLA degradation took place even in the presence of an antioxidant. However, the contrary effect was reported by Fukushima *et al.* [6]. They found a significant stabilizing effect blending PLA with C30B, showing an increase of *T*_5%_ and *T*_max_ in comparison to its blank. Although there are several works regarding PLA nanocomposite thermal degradation, there is a lack of congruence in the results found on the thermal behavior of PLA nanocomposites. In this study, earlier degradation on nanocomposites occurred in comparison with blank PLA, which can be attributed to the presence of C30B, which decreases the activation energy of depolymerization reactions in thermal degradation.

#### 3.2.2. Differential Scanning Calorimetry (DSC)

**Figure 3:**
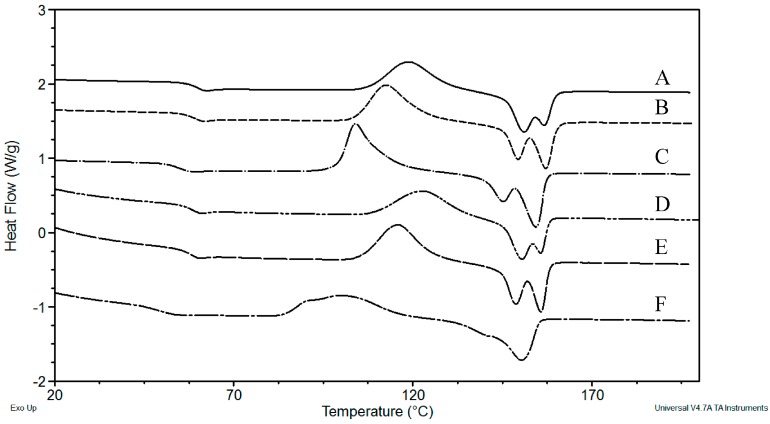
Differential Scanning Calorimetry (DSC) curves of PLA/B (*M*_n_ in kg·mol^−1^ of A = 49.0, B = 33.1, and C = 16.4) and PLA/C30B (*M*_n_ in kg·mol^−1^ of D = 43.5, E = 31.9 and F = 11.3).

The glass transition (*T*_g_), cold crystallization (*T*_c_), and melting (*T*_m_) temperatures of PLA/B (*M*_n_ in kg·mol^−1^ of A = 49.04, B = 33.06, C = 16.40) and PLA/C30B (*M*_n_ in kg·mol^−1^ of D = 43.49, E = 31.90, F = 11.32) recorded after cooling and on a second heating cycle by DSC are shown in Figure 3.

Table 3 reports the thermal events, crystallization enthalpy (*H*_c_), and crystallinity (*X*_c_) of PLA/B and PLA/C30B with different *M*_n_.

It can be observed that *T*_g_ on both PLA/B and PLA/C30B decreases as *M*_n_ decreases. According to Rasselet *et al.* [23], a *T*_g_ decrease can, without any doubt, be attributed to chain scission occurring during oxidation. Indeed, in the case of linear polymers such as PLA, *T*_g_ and *M*_n_ are directly related by the Fox–Flory relationship [24]. When the PLA temperature is below its *T*_g_, large-scale molecular motion is not possible because the material is essentially frozen; after this temperature (on heating), the glassy state changes into rubbery on melt state. *T*_g_ decreased from 59.86 to 55.08 °C in PLA/B, and from 58.27 to 48.49 °C in PLA/C30B as M_n_ diminished. Similar to *T*_g_, *T*_m_ decreased *ca.* 2 °C in PLA/B and *ca.* 5 °C in PLA/C30B in the lowest *M*_n_. However, the reduction of *M*_n_ did not noticeably affect the crystallization of PLA blank because longer chains require more kinetic energy to crystallize. An increase of *X*_c_ (5%) was evidenced in the lowest *M*_n_. Additionally, the addition of C30B in the PLA matrix led to a major increase of *X*_c_ (8%) in nanocomposites with the lowest *M*_n_ in comparison with nanocomposites with the highest *M*_n_. This may be due to the presence of C30B and short chains, which, together, can originate segmental motions at the PLA/C30B interface, allowing PLA crystallization [22]. In semi-crystalline PLA, chain conformation and developed crystallinity are sensitive to surface interactions. It is possible, in the case of nanocomposites, that the high surface interaction between the polymer chains and the nanoclay surface tends to change the degree of crystallinity because of a heterogeneous nucleation effect. Two overlapping melting processes at *ca.* 153 °C are shown in both PLA/B and PLA/C30B, which may correspond to different crystals sizes. The shoulder almost disappears in nanocomposites with the lowest *M*_n_, which may indicate that only one size of crystal remained. It is worth mentioning that similar *X*_c_ was observed as the M_n_ decreased to the lowest value in both blank PLA and nanocomposite. *M*_n_ has minimal effects on Δ*H*_c_; however, *T*_c_ decreased *ca.* 15 °C and *ca.* 26 °C in PLA/B and PLA/C30B, respectively. This indicates that longer PLA chains require higher kinetic energy to break down intermolecular bonds, while the free energy of crystallization of PLA remains. When nanoparticles are introduced into the organic polymer matrix, the polymorphic crystalline form would be induced, giving the nanocomposites many excellent properties, mainly including rapid crystallization [25]. The latter results can be supported by the results of Young *et al.* [26] in their research of PLA morphology and crystallization—they reported that as *T*_c_ decreases, the PLA spherulite radius also decreases from a value of 20 μm at a *T*_c_ of 110 °C to a spherulite radius of 160 μm at a *T*_c_ of 140 °C.

#### 3.2.3. X-ray Diffraction (XRD)

**Figure 4:**
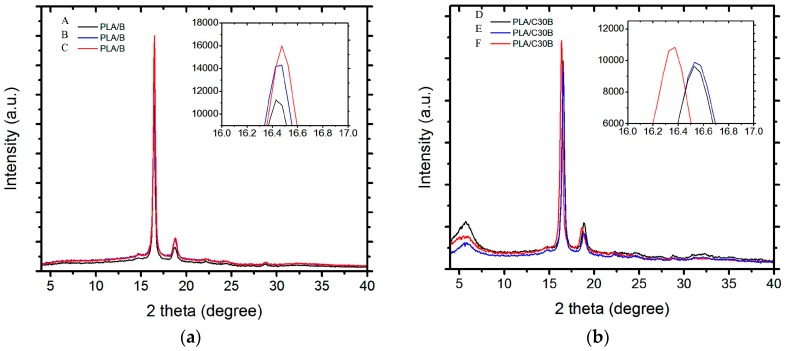
X-ray diffraction (XRD) diffractograms of (**a**) PLA/B (*M*_n_ in kg·mol^−1^ of A = 49.0, B = 33.1, and C = 16.4); and (**b**) PLA/C30B (*M*_n_ in kg·mol^−1^ of D = 43.5, E = 31.9, and F = 11.3).

The X-ray diffraction patterns of PLA/B and PLA/C30B at 0, 176, and 360 h of exposure to artificial weathering are shown in Figure 4. In both blank and nanocompounded PLA (Figure 4a), the diffractogram showed a broad intense peak around 16.5° and a small peak located around 18.5°. These peaks are characteristic of PLA, which is in agreement with the results of dos Santos *et al.* [27] and Liu *et al.* [28]. Nanocompounded PLA (Figure 4b) showed a broad peak at 5.7°, which corresponds to C30B. The difference in intensity in each diffractogram is due to the increase in the crystallinity of PLA at different artificial weathering exposure times. As can be seen from both diffractograms, the intensity of PLA/B and PLA/C30B increases as the artificial weathering exposure time augments. These results are in good agreement with those obtained by DSC (Section 3.2.2.). Chain scission of PLA due to artificial weathering, together with C30B reinforcement favoring the formation of orderly polymer chains, exhibits the development of enhanced crystallinity, and therefore an increase of *X*_c_ (%).

Also, a small peak shift from 16.5° to 16.35° was observed in PLA/C30B at 360 h of exposure to artificial weathering. This may be due to a decrease of the size of the crystal, as previously discussed in Section 3.2.2. From DSC results, it could be observed that the *T*_m_ shoulder in PLA/C30B with the lowest *M*_n_ almost disappears, which may indicate that only one crystal size remained.

#### 3.2.4. Dynamic Thermo-Mechanical Analysis (DTMA)

An essential element in biodegradable applications of polymers is the development of physical and mechanical properties without leaving aside the degradation rate of these polymers. The degradation rate is governed by different factors, such as the nature of the polymer, environmental conditions, and the *M*_W_. For instance, *M*_W_ has a direct correlation with the rate of degradation. Therefore, the design of biodegradable products with the best mechanical properties and lowest *M*_W_ is imperative in order to ensure their short-term degradation.

**Figure 5:**
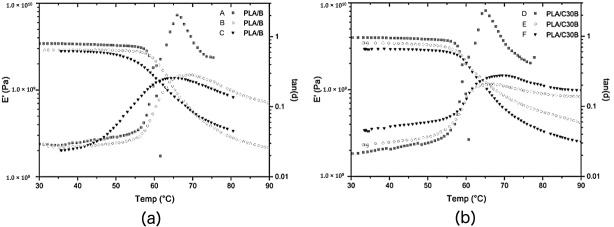
Results of Dynamic Thermo-Mechanical Analysis (DTMA): Storage modulus (*E*′) and tan δ of (**a**) PLA/B (*M*_n_ in kg·mol^−1^ of A = 49.0, B = 33.1, and C = 16.4) and; (**b**) PLA/C30B (*M*_n_ in kg·mol^−1^ of D = 43.5, E = 31.9, and F = 11.3).

Figure 5 shows the *E*′ and tan δ of PLA blank and its nanocomposites as a function of temperature. The curve of both PLA/B and PLA/C30B demonstrates that PLA exhibits glassy, glass-transition, and rubbery behaviors. Also, the curves of PLA/B samples have a similar shape to PLA/C30B along the temperature range from 30 to 90 °C.

Table 4 gives a summary of *E*′ and *T*_g_ of PLA/B and PLA/C30B, both with different *M*_n_ after artificial weathering.

The bonds or forces that keep PLA atoms or chains together are responsible for the viscoelastic properties of PLA. Absorption of the elastic energy into the PLA structure is followed by its dissipation to the structural elements and chemical bonds from the main chain. *E*′ was found to be 3.43 GPa for blank and 3.97 GPa for nanocomposites, both with the highest *M*_n_ (49.04 and 43.49 kg·mol^−1^, respectively). The incorporation of C30B into PLA matrix caused an increment in *E*′ value (increment of 13%) on the highest *M*_n_, indicating that the modulus of PLA improves in the presence of C30B. This may be due to the fact that clay nanolayers restrict the mobility of the surrounding matrix chains and limit the plastic deformation of PLA; thus, stronger interactions between PLA and nanolayers of C30B are obtained. Nanocomposite with a *M*_n_ of 31.90 kg·mol^−1^ had the same *E*′ increment. However, nanocomposites with the lowest *M*_n_ (11.32 kg·mol^−1^) showed a small improvement in *E*´ with respect to its blank (increment of 5%), but still had a better *E*′ (2.95 GPa) than blank PLA (*E*′ = 2.89 GPa) with an *M*_n_ of 33.06 kg·mol^−1^; this may be associated with the major effect of PLA/C30B interactions rather than *M*_n_ decay.

**Figure 6:**
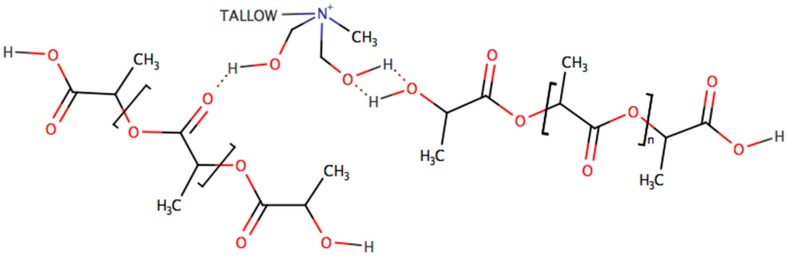
Suggested interactions between the C30B organomodifier and PLA.

PLA has three main functional groups: ester, hydroxyl, and carboxyl groups. On the other hand, C30B organomodifier contains hydroxyl groups that interact with functional groups of PLA via hydrogen bonding, as shown in Figure 6. As the *M*_n_ was reduced, more carboxyl and hydroxyl groups of PLA formed during photo-oxidative chain scission, which means that there were more interaction sites between PLA and the C30B organomodifier, improving PLA crystallization.

The aforementioned suggests that filler interactions among functional groups of PLA and hydroxyl groups of the C30B organomodifier had a major effect on *E*′ improvement rather than *M*_W_ decay. It could also be observed that *E*′ of all PLA samples dropped after the *T*_g_. In semi-crystalline PLA, chain conformation and developed crystallinity are sensitive to surface interactions. That is why in nanocomposites, where a high surface interaction exists between PLA chains and C30B at the nanorange, nanocomposites tend to change their degree of crystallinity and rate of crystallization because of a heterogeneous nucleation effect. The *M*_W_ affects the degree to which internal strains form and recover. Because *E*′ is related to the amount of stored energy during deformation, it provides an estimate of the degree of recovery of PLA. It has been found that the interactions of intercalated and/or exfoliated nanolayers may restrict the mobility of the PLA chains, which lead to an increase of *T*_g_ values, as observed by Yang *et al.* [29] T_g_ was established at the highest tan δ value. Thus, DTMA allows us to study the variations in these parameters linked to macromolecular motions, not only the main one (*T*_g_), but also the local movements not detected by DSC. However, *T*_g_ absolute value obtained by DSC and DTMA cannot be compared. PLA consisted of linear chain molecules having strong intramolecular bonds but weak intermolecular bonds. As the size of PLA chains was reduced (*M*_n_ values lower than 33.1 kg·mol^−1^), mechanical damping was significantly improved, which was directly related to *T*_g_.

#### 3.2.5. Fourier Transform Infrared (FTIR) Spectroscopy

**Figure 7:**
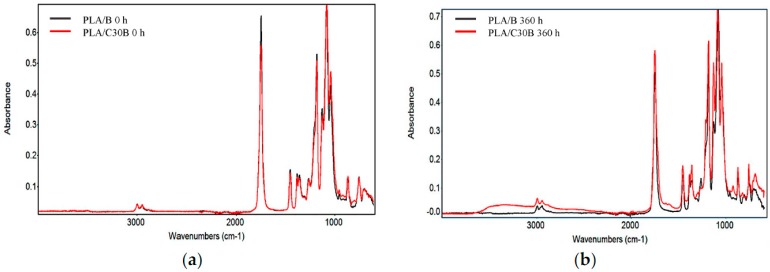
IR spectra of (**a**) PLA/B and PLA/C30B (0 h of exposure to artificial weathering) and; (**b**) PLA/B and PLA/C30B (*M*_n_ in kg·mol^−1^ of D = 43.5, E = 31.9, and F = 11.3).

Several studies have reported that the miscibility of polymer blends can be evaluated appropriately by monitoring the changes in IR vibrational frequency of carbonyl at 1760 cm^−1^ [30] and hydroxyl groups at 3760–3010 cm^−1^ [31]. As a result of an increase in *E*’, and partial exfoliation of C30B into the PLA matrix to form PLA/C30B composites at nanorange, we suggest that intermolecular hydrogen bonds may exist in nanocomposites (see Figure 6). To further confirm this, ATR-FTIR analysis of PLA/B and PLA/C30B at the three artificial weathering exposure times (0, 176, and 360 h) was performed. Figure 7a depicts the FTIR spectra of PLA nanocompounded with 5 wt % load of C30B (PLA/C30B), and blank PLA (PLA/B) at 0 h of exposure to artificial weathering.

The PLA spectrum of both PLA/B and PLA/C30B at 0 h of exposure to artificial weathering shows –CH_3_ stretching at 2994.9–2942 cm^−1^. Also, –C=O stretching at 1747.6 cm^−1^ and –O–C=O stretching at 1176.8–1079.4 cm^−1^ are displayed in both PLA/B and PLA/C30B, which are the main peaks of ester bonds.

Comparison between PLA/B and PLA/C30B at 176 and 360 h of exposure to artificial weathering were carried out in order to identify the source of the enhanced *E*’ (described in Section 3.2.4.). The IR spectrum of PLA/B and PLA/C30B at 176 h is not shown here because they displayed a similar spectrum to PLA/B at 0 h. However, the ATR-FTIR spectrum of PLA/B and PLA/C30B at 360 h of exposure to artificial weathering is shown in Figure 7b. The presence of the stretching frequency of hydroxyl groups (3600–3050 cm^−1^) was found in PLA/C30B at 360 h of exposure to artificial weathering, which is one of the basic IR characteristics of the formation of hydrogen bonds. According to Orozco *et al.* [30], the broad band in the range of 3760–3010 cm^−1^ indicates hydrogen-bonded hydroxyl groups in starch. It seems that hydroxyl in C30B organomodifier gets involved in the formation of intermolecular hydrogen bonding with PLA matrix, as previously explained in Figure 6. However, since the amount of C30B was only 5 wt % in PLA/C30B blends, and also because only about one third of total mass corresponds to the amount of organomodifier, the –OH stretching of PLA vibrational frequency is very weak.

An enlargement of the carbonyl peak at 1745 cm^−1^ was also observed. In some studies [32], a semicrystaline polymer with a –C=O of 1747 cm^−1^ has been blended with another semicrystalline polymer of Polyhydroxybutyrate (PHB) with a –C=O of 1714 cm^−1^. The weak interaction between the carbonyl groups and hydrogen atoms of both matrices through hydrogen bonding leads to a miscible blend, which results in an overlaid C=O peak in the FTIR spectra of PLA/C30B blend. The low vibrational frequency of –C=O may be ascribed to the formation of intermolecular hydrogen bonds.

## 4. Conclusions

In this study, Cloisite30B dispersed at nanorange throughout the PLA matrix was used in order to investigate the thermo-mechanical properties of PLA nanocomposites with different *M*_W_.

The *M*_n_ values of PLA blank and nanocomposites showed decay due to processing before exposure to artificial weathering. The *M*_n_ of irradiated PLA/B and PLA/C30B decreased notably in comparison with original PLA; this clearly showed a depolymerization of PLA by artificial weathering.

Thermal treatments of C30B were performed in order to study the thermal degradation of organomodifier during nanocomposite processing. The results showed that the weight loss during processing of the organomodifier was negligible. In addition, C30B alone was subjected to the same three artificial weathering exposure times (0, 176, and 360 h) as blank and nanocomposites without observing significant degradation of the organomodifier due to photodegradation. The content of organomodifier of C30B (amounting to about one third of the total mass) remains available to allow interactions with PLA.

The thermal stability of the PLA matrix was not improved by the presence of C30B in comparison with PLA/B in either of the samples with different *M*_W_. On the contrary, it could be observed that *T*_g_ obtained by DSC of PLA decreased as the *M*_n_ decreased, which may be attributed without any doubt to chain scission during artificial weathering degradation.

On the other hand, *T*_g_ obtained by DTMA analysis increased as *M*_n_ decreased due to the amount of stored energy during deformation that was related to *E*′ improvement (increment of 13%). It has been found that the interactions of exfoliated nanolayers may restrict the mobility of the PLA chains, which led to an increase of *E*′ and *T*_g_ values. We suggest that filler/polymer interactions are still present between the functional groups of PLA and the hydroxyl groups of C30B organomodifier, even at the longest artificial weathering exposure time, which led to a major E′ improvement due to hydrogen bonding.

## Figures and Tables

**Table 1:** Molecular weight of samples.

Sample	Exposure Time (h)	*M*_n_ (kg·mol^−1^)	*M*_W_ (kg·mol^−1^)	*M*_n_/*M*_W_
PLA neat	–	135.9	329.0	2.42
PLA/B	0	49.0	125.0	2.55
	176	33.1	74.3	2.25
	360	16.4	33.3	2.03
	0	43.5	99.0	2.27
PLA/C30B	176	31.9	69.7	2.18
	360	11.3	22.8	1.72

PLA: Polylactide; PLA neat: Original PLA sample; PLA/B: PLA blank; PLA/C30B: Polylactide/Cloisite30B nanocomposite.

**Table 2:** Decomposition temperature at 10%–90% of weight loss.

PLA/B (Temperature °C)				PLA/C30B (Temperature °C)		
Weight loss (%)	*M*_n_ 49.04 kg·mol^−1^	*M*_n_ 33.06 kg·mol^−1^	*M*_n_ 16.40 kg·mol^−1^	*M*_n_ 43.49 kg·mol^−1^	*M*_n_ 31.90 kg·mol^−1^	*M*_n_ 11.32 kg·mol^−1^
10	334.81	329.57	318.05	328.77	313.80	307.60
20	344.94	341.42	336.15	340.99	332.84	323.65
30	350.69	348.06	344.94	347.77	341.89	334.43
40	355.03	352.93	350.65	352.6	347.68	342.34
50	358.70	357.01	355.18	356.55	352.16	348.42
60	361.95	360.68	359.01	360.02	355.99	353.50
70	364.81	364.22	362.51	363.31	359.45	358.23
80	367.45	367.93	366.20	366.96	363.13	363.28
90	371.42	372.38	371.42	372.72	369.33	370.93

**Table 3:** Thermal events and crystallinity of PLA blank and nanocomposites.

PLA/B						PLA/C30B					
*M*_n_ (kg·mol^−1^)	*T*_g_ (°C)	*T*_c_ (°C)	*T*_m_ (°C)	Δ*H*_c_ (J·g^−1^)	*X*_c_ (%)	*M*_n_ (kg·mol^−1^)	*T*_g_ (°C)	*T*_c_ (°C)	*T*_m_ (°C)	Δ*H*_c_ (J·g^−1^)	*X*_c_ (%)
49.0	59.86	119.13	156.83	35.96	22.75	43.5	58.27	122.77	155.85	33.14	18.92
33.1	59.22	112.66	157.32	37.52	23.93	31.9	58.07	115.72	155.86	38.24	23.23
16.4	55.08	104.02	154.56	38.16	27.75	11.3	48.49	100.12	150.46	39.13	27.01

**Table 4:** Mechanical properties of PLA blank and nanocomposites.

Sample	*E*′ (GPa)	*T*_g_ (° C)
PLA/B (*M*_n_ 49.04 kg·mol^−1^)	3.43	65.95
PLA/B (*M*_n_ 33.06 kg·mol^−1^)	2.89	69.88
PLA/B (*M*_n_ 16.40 kg·mol^−1^)	2.82	65.27
PLA/C30B (*M*_n_ 43.49 kg·mol^−1^)	3.97	58.28
PLA/C30B (*M*_n_ 31.90 kg·mol^−1^)	3.43	66.51
PLA/C30B (*M*_n_ 11.32 kg·mol^−1^)	2.94	69.43

## Abbreviations

ATR-FTIR	Attenuated Total Reflectance-Fourier Transform Infrared
C30B	Cloisite30B
DMTA	Dynamic Mechanical Thermal Analysis
DSC	Differential Scanning Calorimetry
GPC	Gel Permeation Chromatography
HPLC	High Performance Liquid Chromatography
MMT	Montmorillonite
*M*_W_	Molecular Weight
*M*_W_ D	Molecular Weight Distribution
*M*_W_	Weight Average Molecular Weight
*M*_n_	Number Average Molecular Weight
PLA	Poly-Lactide
PLA/B	Poly-Lactide blank
PLA/C30B	Poly-Lactide nanocomposite
TGA	Thermogravimetric Analysis
THF	Tetrahydrofuran
*T*_g_	Glass Transition Temperature
*T*_c_	Cold Crystallization Temperature
*T*_m_	Melting Temperature
*X*_c_	Degree of Crystallinity
XRD	X-ray Diffraction
